# The impact of self-efficacy on the well-being of primary school teachers: a Greek-Cypriot study

**DOI:** 10.3389/fpsyg.2023.1223222

**Published:** 2023-10-19

**Authors:** Glykeria Reppa, Marilena Mousoulidou, Eirini Tzovla, Christiana Koundourou, Andri Christodoulou

**Affiliations:** Department of Psychology, School of Health Sciences, Neapolis University Pafos, Paphos, Cyprus

**Keywords:** well-being, self-efficacy, PERMA, Greece, Cyprus

## Abstract

**Introduction:**

This research was conducted to examine the extent to which teachers’ self-efficacy affects their well-being following the COVID-19 pandemic. The originality of the research lies in the fact that there are not enough studies that simultaneously examine the relationship between well-being and self-efficacy in primary school teachers in Greece and Cyprus.

**Methods:**

A total of 258 primary school teacher participants took part in this study, aged 23–62. The Teachers’ Sense of Efficacy Scale and the PERMA Profiler questionnaire were used to study the relationship between teachers’ well-being and self-efficacy.

**Results:**

Results show that after the COVID-19 pandemic, teachers’ well-being is moderate to high. Moreover, teachers’ self- efficacy is also high and related to their well-being.

**Discussion:**

An important finding from the current research is that teachers’ self-efficacy in promoting student engagement was the most important predictor for teachers’ well-being. The implications of the results are discussed, and recommendations are made.

## 1. Introduction

The profession of the teacher is associated with a variety of demands derived from factors arising from technology, the formulation of educational programs, and the formulation of specific individual targets for each student (Arvidsson et al., 2019). In addition, teachers are required to participate in different administrative and parent meetings, deal with the administrative bureaucracy, prepare the teaching material, and manage increasingly more diverse classrooms where multicultural learning and discipline must be maintained (Jomuad et al., 2021). Moreover, teachers are required to manage many changes, such as time pressure, challenging relationships with coworkers and administrative personnel, bad working conditions, the ambiguity of the role of the teacher, and job insecurity (Shilpa, 2021). All the above make them feel ineffective in managing students’ behavior in the classroom and affect the satisfaction they derive from their profession and their self-efficacy (Sulla and Rollo, 2023).

Furthermore, the sudden appearance of the COVID-19 pandemic led to many challenges to the educational environment and new needs for teachers, such as increased fatigue due to novel working conditions and remote distance learning (Bartosiewicz et al., 2022; Shimony et al., 2022). Teachers were faced with a variety of challenges, such as remote-modern teaching methods, the lack of supporting school frameworks, the lack of knowledge for the proper utilization of technology and learning with the help or participation of the parents (Kasprzak and Mudlo-Glagolska, 2022; Shimony et al., 2022). These challenges appear to have an impact on teachers’ well-being (Bartosiewicz et al., 2022; Shimony et al., 2022). A predictive factor that seems to enhance teachers’ ability to cope with professional difficulties is their self-efficacy (Baka, 2017; Sulla and Rollo, 2023), as is investigated in the current research.

## 2. Literature review

### 2.1. Well-being

Well-being concerns all of us and affects our daily lives (De Stasio et al., 2017). However, although it is often referred to as well-being, this concept is subjective and difficult to define (Soutter et al., 2014; McCallum et al., 2017). It is a psychological construct that refers to happiness, positivity, and resilience (Wigford and Higgins, 2019) and includes a feeling of wellness, where the person tends to feel happier, more devoted, more compassionate, understanding, and grateful (O’Brien and Guiney, 2021). Moreover, well-being is associated with positive emotions, perceived social support, and meaning in life (Pezirkianidis et al., 2021). It refers to a healthy emotional and physical situation with adequate emotional, cognitive, and behavioral resources to respond to difficult circumstances or conditions (Wigford and Higgins, 2019; O’Brien and Guiney, 2021).

Similarly, due to the complexity of the concept of well-being, it is difficult to provide a specific definition for teachers’ well-being, and as such several definitions have been formulated (Cann et al., 2020). According to Viac and Fraser (2020), teachers’ occupational well-being is a multidimensional concept, which is defined as “teachers’ responses to the cognitive, emotional, health and social conditions pertaining to their work and their profession” (p. 18). This plays an important role in the sustainability of the teachers’ profession, and those who make educational policies should ensure that the well-being of teachers is maintained high in their workplace (McCallum et al., 2017). When teachers’ well-being is high, it seems to be related to the effective management of the classroom (Sulla and Rollo, 2023), promoting supportive relationships between teachers and students, enhancing students’ well-being, and reducing their psychological stress, cultivating emotional intelligence, and promoting social learning (Hoglund et al., 2015; Harding et al., 2019; Jennings et al., 2019). Teachers’ well-being is enhanced when they feel that they are closer to their everyday work life and attribute a positive meaning to it, resulting in improved job satisfaction (Brady and Wilson, 2021; O’Brien and Guiney, 2021). Α positive working environment, appreciation of teachers’ work and effort, good communication among coworkers, and a higher sense of belonging in a supportive school, are central pillars of positive teachers’ well-being (Cann et al., 2020; Skinner et al., 2021), which influence not only the teachers’ life but also positively motivate students and shape a good learning climate (De Stasio et al., 2017).

The interest in well-being has increased considerably in recent years (Cann et al., 2020), and that is why the Organization for Economic Co-operation and Development (OECD) recommended instruments to measure teachers’ occupational well-being (Viac and Fraser, 2020). Such instruments are the Satisfaction with Life Scale (Diener et al., 1985), which assesses satisfaction as a whole, the Subjective Happiness Scale (Lyubomirsky and Lepper, 1999), which assesses whether a person is happy or unhappy, and the PERMA Profiler (Butler and Kern, 2016) which will be used in the current research.

This instrument is based on one of the most influential theories that examine the overall sense of well-being and its elements, the PERMA theory (Seligman, 2011). This theory supports that there are five components of individual psychological flourishing. These components form the acronym of the name of the measure PERMA; that is, Positive Emotion, Engagement, Relationships, Meaning, and Accomplishment. The first component, Positive Emotions, involves experiencing positive emotions in everyday life; that is being able to focus on positive emotions such as happiness, comfort, and optimism and being able to view the past, present, and future in a positive way. According to Frederickson (2003), these feelings can be taught and developed. The second component, Engagement, includes being able to experience the psychological flow, be engaged, and feel satisfied with everyday activities. Engagement in everyday activities is important as it helps individuals learn, mature and foster their personal happiness. The component of Relationships involves feeling supported, accepted, and loved by an individual’s social network and helps individuals build positive relationships. Positive and strong relationships involve trusting others and giving or receiving support from social networks when it is needed, especially through difficult times. The fourth component, Meaning, concerns the feelings and beliefs of individuals that life is important and the importance of living a meaningful life and having a sense of purpose. When individuals are high in Meaning, they seek to find the real meaning in life instead of searching for pleasure and material wealth. The last component, Accomplishment, involves individuals’ satisfaction when they are able to accomplish their personal goals (Seligman, 2011; Symeonidou et al., 2019; Pezirkianidis et al., 2020, 2021). The PERMA Profiler provides both separate scores for each component and an overall well-being score (Pezirkianidis et al., under review).

In the literature, there are only a few studies that used the PERMA Profiler to examine teachers’ well-being. However, these studies showed that high levels of teachers’ well-being appear to have a significant positive effect in a number of domains (Turner et al., 2021). In a recent phenomenological study of Australian teachers’ well-being, carried out by Turner and Thielking (2019), teachers stated feeling less stressed, calmer, and more positive while teaching when they intentionally used positive psychology strategies in their everyday teaching practices.

Kun and Gadanecz (2019) in their mixed method study of 300 Hungarian teachers used the PERMA Profiler to measure teachers’ well-being and happiness within their workplace. Their research concluded that when teachers perceive their work in a meaningful way, engage in positive workplace relationships, and when there is an overall positive workplace climate, their perception of workplace happiness and well-being is affected positively. Furthermore, their study concluded that all dimensions of PERMA well-being, the psychological factors of hope, self-efficacy, resilience, and optimism, were positively related to workplace happiness; a finding also supported by Kun and Gadanecz (2019). Zeng et al. (2019), in their correlation analysis study of 471 Chinese secondary school teachers, found a positive association with mindset, perseverance of effort, and work engagement when they used the PERMA profiler to measure teachers’ well-being.

### 2.2. Self-efficacy

Self-efficacy is a concept mentioned by many theorists and researchers (Gibson and Dembo, 1984; Bandura, 1997; Tschannen-Moran et al., 1998) and concerns a person’s self-perceptions of their teaching ability (Tschannen-Moran et al., 1998). Rotter’s Theory of Locus of Control (Tschannen-Moran et al., 1998) is a forerunner of self-efficacy theory, but this concept was extensively developed by Bandura (1997) in his Social Learning Theory. Self-efficacy, according to Bandura (1997, 2006), is defined as “beliefs in one’s capabilities to organize and execute the courses of action required to produce given attainments” (p. 3). According to the theory, self-efficacy is formed by four sources: (a) mastery experiences, (b) vicarious experience, (c) verbal persuasion, and (d) physiological and affective states. It concerns personal self-efficacy, which is the “personal value, ability, and effort that the individual puts into achieving a goal in relation to a certain condition of the environment” (Tzovla and Kedraka, 2023, p. 153), and outcome expectancy, which is the “estimation of the individual that a given behavior will lead to specific results” (Bandura, 1997, p. 193).

Gibson and Dembo (1984) used Bandura’s theory and identified two types of teachers’ self-efficacy. The first is Personal Teaching Efficacy (PTE), which corresponds to personal self-efficacy. The second is General Teaching Efficacy (GTE), which corresponds to outcome expectancy. Tschannen-Moran et al. (1998) define it as “the teacher’s belief in his or her capability to organize and execute courses of action required to successfully accomplish a specific teaching task in a particular context” (p. 233) and note the importance of the active involvement of students including those with difficulties (Tschannen-Moran and Woolfolk Hoy, 2001).

Teachers’ self-efficacy is a subjective concept shaped by the teachers’ interaction with their environment and affects their expectations, motivations, goals, self-regulation, educational behavior, teaching practices, attitudes towards the various subjects, and learning outcomes (Gibson and Dembo, 1984; Riggs and Enochs, 1990; Bandura, 1997; Shahzad and Naureen, 2017). Moreover, high self-efficacy is related to adopting innovation in teaching practice, increased support, encouragement, and autonomy of students (Caprara et al., 2006), and strengthening parental involvement in the educational process (Egyed and Short, 2006). Attending teacher professional development programs seems to positively affect its improvement (Aji and Khan, 2019; Mertasari and Candiasa, 2020; Tzovla and Kedraka, 2021; Tzovla et al., 2021a,b). In addition, teachers’ self-efficacy is documented to influence students’ self-efficacy (Trygstad et al., 2014), their behavior, and their performance (Shahzad and Naureen, 2017).

Note that “teachers do not feel equally effective in all teaching situations” (Goddard et al., 2000, p. 482), and given that self-efficacy concerns a specific task (Bandura, 1997, 2006), the measurement instruments of self-efficacy differ depending on the field of action and the target group (Bandura, 1997). For this reason, a number of tools have been constructed, such as the Teacher Efficacy Scale (TES) (Gibson and Dembo, 1984), which measures personal teaching efficacy and general teaching efficacy; the Teachers’ Sense of Efficacy (TSES) (Tschannen-Moran and Woolfolk Hoy, 2001), which measures three parameters of the learning process teaching strategies, classroom management and student engagement; the Teachers’ Efficacy Beliefs System-Self (TEBS-Self) (Dellinger et al., 2008) which measures the level of teachers’ self-efficacy regarding tasks related to effective teaching and learning in their classroom context; the Science Teaching Efficacy Belief Instrument (STEBI A and B) (Riggs and Enochs, 1990) which measures self-efficacy of in-service and pre-service elementary school teachers in Sciences.

### 2.3. Well-being and self-efficacy

Billett et al. (2023), in their research with 534 teachers in Australia during the COVID-19 pandemic, indicated that teachers’ well-being is positively related to self-efficacy. Self-efficacy was found to be associated with establishing classroom management systems and noted that teaching experience and age affect self-efficacy, with teachers who had been teaching for 15–19 years and aged 60–70 years having the highest self-efficacy. Moreover, Soykan et al. (2019), in a study with 1,502 teachers in New Zealand, found a positive relationship between teachers’ well-being and self-efficacy. Arslan (2018), in a study with 295 Turkish educators, found a positive relationship between the well-being and self-efficacy of teachers and vice versa. Worth noting that the relationship between self-efficacy and well-being has also been studied in relation to other aspects, such as job satisfaction and years of teaching experience (Lauermann and König, 2016; Toropova et al., 2020; Bartosiewicz et al., 2022), low student motivation, and lack of supervisory support (Skaalvik and Skaalvik, 2016) among others.

### 2.4. Purpose of the study – hypotheses

The aim of the current study was to examine the influence of self-efficacy on the well-being of Greek and Cypriot primary. Teachers are required to hold a Bachelor’s degree in Primary Education in order to work in schools. Placement in private schools follows an independent application and interview process prior to employment, whereas placement in public schools requires teachers to enlist in the hiring catalog of the Ministry of Education, which is responsible for their employment (Eurydice, 2022). The process of entering public primary schools is time-consuming and may take a few years before teachers are placed. As such, many further their qualifications to attain a higher ranking in the catalog and ensure placement. Throughout their teaching career, teachers are mandated to participate in educational seminars and professional development programs in order to enhance their knowledge and skills in order respond to the ever-increasing pedagogical demands of their profession (Tzovla et al., 2021a).

Although the impact of self-efficacy on well-being is well documented in the literature, this research is significant due to the period it was conducted. Specifically, the current research took place during March and April 2023, a time when the education sector was returning to “normality” after the significant changes that took place because of the COVID-19 pandemic. It is well documented in the literature that the changes caused by the pandemic created a lot of challenges for teachers at all levels (i.e., primary, secondary, tertiary) that inevitably negatively influenced their well-being. Research around the globe has shown that because of the changes that took place during the pandemic, such as changes in the way lessons were delivered (e.g., online, hybrid), affected teachers’ well-being, with several teachers showing signs of stress, anxiety, and even depression (see Kang et al., 2022, for a review). Similar results were also obtained from studies examining Greek-speaking participants. For instance, Papazis et al. (2022) found higher stress levels in primary school teachers during the COVID-19 pandemic lockdown. Similarly, Raikou et al. (2021) found low self-esteem and medium anxiety in primary and secondary school teachers. Stachteas and Stachteas (2020) found that one-third of their secondary school teachers’ sample was feeling very anxious. Lastly, in a large study with 1,157 participants that included teachers, parents, and adolescents, Hatzichristou et al. (2021) found that teachers had a high tendency for anxiety, but importantly teachers also had high levels of coping with it. All of the above research was conducted during the period that the COVID-19 pandemic was influencing the education sector because of the different measures that took place to stop the spread of the virus, such as school closures and lockdowns. To our knowledge, no other study examined Greek and Cypriot primary school teachers’ well-being after the end of these measures. It was therefore considered imperative to conduct this study for a more comprehensive view of the well-being of teachers now that the pandemic is coming to an end.

Based on this aim and the existing literature, the following hypotheses were formulated:

*H1*: There will be a positive association between self-efficacy and well-being. Following previous research findings, we expected that the greater teachers’ self-efficacy, the higher their well-being.

*H2*: There will be differences between demographics and all variables of interest (well-being and self-efficacy). This hypothesis was more exploratory in nature, and therefore no clear predictions were made.

## 3. Methods

### 3.1. Participants

A total of 258 primary school teacher participants took part in this study. The vast majority of the sample had a Greek nationality (77.9%), while the rest had a Cypriot (22.1%) nationality. The age of the sample ranged between 23 and 62. Namely, 85 participants (32.9%) were young-adults (23–39 years old), 93 participants (26%) were young-middle-aged adults (40–51 years old), and 80 participants (31%) were late-middle-aged adults (52 to 62 years old). Concerning education level, more than half of the participants (147 participants, 57%) had a Master’s degree, followed by 89 participants (34.5%) who had a Bachelor’s degree, and 22 participants (8.5%) had a Doctorate degree. Two hundred and twenty-four participants (86.8%) were employed in the public sector, and 34 participants (13.2%) in the private sector. Regarding years of experience, 121 participants (46.9%) had more than 20 years of experience, 45 participants (17.4%) had 16–20 years of experience, 38 participants (14.7%) had 11–15 years of experience, 18 participants (7%) had 6–10 years of experience, and 36 participants (14%) had 0–5 years of experience. For administrative duties, almost half of the participants (105 participants, 40.7%) reported that they never had administrative duties, followed by 69 participants (26.7%) who reported having daily administrative duties, 49 participants (19%) reported having administrative duties once per week, and 35 participants (13.6%) once per month. For other duties, almost half of the participants (119 participants, 46.1%) reported that they had duties once per month, 67 participants (26%) had daily duties, followed by 49 participants (19%) who reported having duties once per week, and 23 participants (8.9%) who reported that they never had other duties.

### 3.2. Measures

#### 3.2.1. Demographic information

A demographic questionnaire was administered, which asked participants to provide their age, gender, nationality, education level, years of experience, employment sector, and administrative and other duties.

#### 3.2.2. Teachers’ sense of efficacy scale

To measure teachers’ self-efficacy, the long version of the Teachers’ sense of efficacy scale (TSES) scale (Tschannen-Moran and Woolfolk Hoy, 2001; Tsingilis, 2005, for the Greek version) was used. This version comprises 24 items which are grouped into three factors. The first factor is Efficacy in using Instructional Strategies. It consists of 8 items and measures teachers’ efficacy in helping students academically (e.g., “How much can you do to adjust your lessons to the proper level for individual students?”). The second factor is Efficacy in Classroom Management, which comprises 8 items and measures teachers’ competence in managing their classroom (e.g., “How well can you keep a few problem students from ruining an entire lesson?”). The last factor is Efficacy in promoting Student Engagement. This factor also consists of 8 items and measures teachers’ efficacy in motivating, building relationships and solving student problems (e.g., “How much can you do to help your students value learning?”). Additionally, the scale provides an Overall Sense of Efficacy by averaging the total of the three scales. Each item is rated on a 9-point Likert-type scale ranging from 1 (Nothing) to 9 (A Great Deal). The items’ internal reliability for each factor of TSES as well as the Overall Sense of Efficacy, were very high and similar to those obtained by Tschannen-Moran and Woolfolk Hoy (2001). For the factors Efficacy in using Instructional Strategies, Efficacy in Classroom Management, and Efficacy in promoting Student Engagement, the Cronbach’s alpha was 0.89 (*α* = 0.91 in Tschannen-Moran and Hoy), 0.96 (*α* = 0.90 in Tschannen-Moran and Hoy), and 0.93 (*α* = 0.87 in Tschannen-Moran and Hoy), respectively. The Cronbach’s alpha for the Overall Sense of Efficacy was 0.97 (α = 0.94 in Tschannen-Moran and Hoy).

#### 3.2.3. PERMA profiler

To measure teachers’ well-being, the PERMA Profiler questionnaire (Butler and Kern, 2016; Pezirkianidis et al., 2021, for the Greek language) was used. The PERMA Profiler comprises 15 questions (3 for each factor) and an additional eight questions that are used as complementary items, which measure Negative Emotions (3 items), Health (3 items), Loneliness (1 item), and Happy (1 item). The measure also provides a score for Overall Well-Being by averaging all 23 items. The factor Positive Emotions measures an individual’s tendency toward feeling joyful, positive, and content (e.g., “In general, how often do you feel joyful?”). Engagement assesses an individual’s absorption, involvement, and interest in activities or generally the world itself (e.g., “How often do you become absorbed in what you are doing?”). The factor Relationships measures an individual’s positive relationships with others for feeling supported, loved, and valued (e.g., “To what extent do you receive help and support from others when you need it?”). Meaning assesses a person’s sense of purpose in life and the value of life itself (e.g., “In general, to what extent do you feel that what you do in life is valuable and worthwhile?”). The factor Accomplishment measures an individual’s sense of accomplishment of goals, responsibilities, and tasks (e.g., “How much of the time do you feel you are making progress towards accomplishing your goals?”). The items’ internal reliability for the factor Positive Emotions and Overall Well-Being was high (*α* = 0.88 for Positive Emotions, *α* = 0.85 for Overall Well-Being), similar to those obtained by Butler and Kern (2016). For the factors Relationships, Meaning, and Accomplishment, the items’ internal reliability was moderate (*α* = 0.75 for all three factors) and for the factor Engagement, low to moderate (*α* = 0.58). These values are lower than those obtained by Butler and Kern.

### 3.3. Procedure

Upon receiving necessary ethical approval from the Cyprus National Bioethics Committee (EEBK EΠ 2023.01.11 18.01.2023), the measures used in this study were combined into a survey created via Google Forms. Data were collected via convenience and snowball sampling methods where teacher participants were asked to disseminate the survey to their colleagues between 23 March 2023 and 7 April 2023. Since this study involved primary school teachers, only the responses of participants working in primary education in Cyprus or Greece were included in the analysis. The questionnaire was distributed to the participants online using email, social chatting apps (e.g., WhatsApp, Viber, Messenger), and social media (e.g., Facebook, Instagram). All participants were contacted online, informed about the purpose and duration of the study, and assured of their anonymity in participating. Their consent to participate in the research was received prior to completing the questionnaire. The completion of the questionnaire took approximately 15–20 min.

### 3.4. Data analysis

All data were entered into the Statistical Package for Social Sciences, Version.

25.0 (SPSS 25, IBM Corporation, Armonk, NY, USA), the level of significance for the tests was set to 5%, and all the necessary analyses were conducted. Normality tests showed that the data were approximately normally distributed, Shapiro–Wilk *p* > 0.005 and Kolmokorov-Smirnov *p* > 0.005. Since our data were normally distributed, we further conducted parametric tests. Descriptive statistics were computed. Subsequently, we conducted a Pearson correlation to examine the relationship between the factors of TSES and PERMA Profiler. Then ANOVAs were computed to compare the effect of age on self-efficacy and well-being. Lastly, all necessary assumptions to run a linear multiple regression were satisfied and it was computed to examine the predictors of well-being among teachers.

## 4. Results

The means and standard deviations of all variables are presented in Table 1. For the variable efficacy, as measured by TSES, participants reported a moderate level for all factors; that is, efficacy in using instructional strategies, efficacy in classroom management, efficacy in promoting student engagement, and overall sense of efficacy. For the variable well-being, measured by PERMA Profiler, results showed a moderate to a high level for all factors (i.e., positive emotion, engagement, relationships, meaning, accomplishment, and overall well-being). These values are comparable to those obtained by the developers of the tools (Tschannen-Moran and Woolfolk Hoy, 2001 for TSES; Butler and Kern, 2016, for PERMA Profiler).

**Table 1:** Mean scores with standard deviations for all variables of interest.

Variables	Sample (*N* = 258) mean scores (SD)	Developers mean scores (SD)
Efficacy (TSES)		
Instructional strategies	7.4 (0.9)	7.3 (1.1)^a^
Classroom management	7.2 (1.2)	6.7 (1.1)^a^
Student engagement	7.2 (1.1)	7.3 (1.1)^a^
Overall sense of efficacy	7.3 (0.9)	7.1 (0.9)^a^
Well-being (PERMA)		
Positive emotions	7.0 (1.8)	6.7 (1.9)^b^
Engagement	7.2 (1.5)	7.3 (1.7)^b^
Relationships	7.3 (1.7)	6.9 (2.2)^b^
Meaning	7.5 (1.5)	7.1 (2.2)^b^
Accomplishment	7.3 (1.4)	7.2 (1.8)^b^
Overall well-being	7.3 (1.3)	7.0 (1.7)^b^

^a^Tschannen-Moran and Woolfolk Hoy (2001), *N* = 111; ^b^Butler and Kern (2016), *N* = 31,965.

Hypothesis 1 was supported. Pearson’s correlation was used to explore possible associations between the three factors of TSES (Instructional Strategies, Classroom Management, Student Engagement) and Overall Sense of Efficacy, and the five factors of PERMA (Positive Emotions, Engagement, Relationships, Meaning, Accomplishment) and Overall Well-Being. The results indicated moderate positive significant relationships between all factors of TSES and all factors of PERMA (Table 2). The higher the efficacy in using instructional strategies, the greater their positive emotions, engagement, relationship, meaning, accomplishment, and overall well-being. Also, the greater teachers’ efficacy in classroom management, the higher their positive emotions, engagement, relationship, meaning, accomplishment, and overall well-being. Moreover, the higher teachers’ efficacy in promoting student engagement, the greater teachers’ positive emotions, engagement, relationship, meaning, accomplishment, and overall well-being. Lastly, the greater teachers’ overall sense of efficacy, the higher their positive emotions, engagement, relationship, meaning, accomplishment, and overall well-being. Worth noting is that high associations were found between the factors of the TSES instrument (ranging from 0.677 to 0.902), as well as between the factors of the PERMA Profiler (ranging from 0.491 to 0.901).

**Table 2:** Correlations between efficacy and well-being.

	1	2	3	4	5	6	7	8	9	10
Efficacy (TSES)										
Instructional strategies	---									
Classroom management	0.667**	---								
Student engagement	0.751**	0.782**	---							
Overall sense of efficacy	0.877**	0.902**	0.927**	---						
Well-being (PERMA)										
Positive Emotion	0.350**	0.454**	0.506**	0.477**	---					
Engagement	0.388**	0.444**	0.501**	0.482**	0.652**	---				
Relationship	0.301**	372**	0.434**	0.404**	0.698**	0.491**	---			
Meaning	0.462**	0.560**	0.601**	0.586**	0.750**	0.598**	0.598**	---		
Accomplishment	0.496**	0.566**	0.632**	0.610**	0.666**	0.656**	0.517**	0.734**	---	
Overall well-being	0.459**	0.557**	0.619**	0.574**	0.901**	0.803**	0.780**	0.861**	0.828**	---

**Correlation is significant at the 0.01 level (two-tailed). 1, instructional skills (TSES); 2, classroom management (TSES); 3, student engagement (TSES); 4, overall sense of efficacy (TSES); 5, positive emotion (PERMA); 6, engagement (PERMA); 7, relationship (PERMA); 8, meaning (PERMA); 9, accomplishment (PERMA); 10, overall well-being (PERMA).

Hypothesis 2 examined the impact of the demographic variables (age, gender, education level, employment sector, years of experience, and administrative and other duties) on the variables of interest (Well-Being and Efficacy). However, the characteristics of our sample were such that only the numbers of the variable age were evenly distributed (see Section 2.2.1). For this reason, analysis was conducted only for this variable.

For the variable age, we first explored the impact of age on well-being as measured by the PERMA Profiler. A one-way ANOVA was computed to compare the effect of the three age groups (young-adults, early-middle-aged adults, and late-middle-aged adults) on the five factors of the PERMA Profiler (Positive emotions, Engagement, Relationships, Meaning, Accomplishment) and Overall Well-Being. The analysis revealed statistically significant differences between age and the PERMA Profiler factor Meaning *F*(2, 255) = 3.76, *p* = 0.025. Tukey’s HSD post-hoc multiple comparisons tests were computed to further explore this significance, as it is considered a good method (Kim, 2015). Post-hoc comparisons indicated that the mean value for the young-adults (*M* = 7.16, *SD* = 1.54) was significantly different from the late-middle-aged adults (*M* = 7.77, *SD* = 1.38). There was no statistically significant difference in mean scores between young-adults and early-middle-aged adults (*p* = 0.16) or between early-middle-aged adults and late-middle-aged adults (*p* = 0.61). There were also statistically significant differences between age and the PERMA Profiler factor Accomplishment [*F*(2, 255) = 7.43, *p* < 0.001]. Tukey HSD post-hoc test showed that the mean value for the young-adults (*M* = 6.92, *SD* = 1.42) was significantly different from the late-middle-aged adults (*M* = 7.74, *SD* = 1.21). There was no statistically significant difference in mean scores between young-adults and early-middle-aged adults (*p* = 0.07) or between early-middle-aged adults and late-middle-aged adults (*p* = 0.19). The analysis for age and Overall Well-Being was also statistically significantly different [*F*(2, 255) = 3.19, *p* = 0.04]. Tukey HSD test, however, showed no statistically significant difference in mean scores between young-adults and early-middle-aged adults (*p* = 0.068), nor between young-adults and late-middle-aged adults (*p* = 0.082), or between early-middle-aged adults and late-middle-aged adults (*p* = 1). There were no statistically significant differences for age group and the factors Positive emotions [*F*(2, 255) = 2.03, *p* = 0.13], Engagement [*F*(2, 255) = 2.82, *p* = 0.061], and Relationships [*F*(2, 255) = 1.87, *p* = 0.16]. The results suggest that, at least for the factors Meaning and Accomplishment, there is a general trend where young-adult teachers’ well-being is different from late-middle-aged teachers.

A one-way ANOVA was also conducted to compare the effect of the three age groups (young-adults, early-middle-aged adults, and late-middle-aged adults) on the three factors of the TSES Profiler (Efficacy in using Instructional Strategies, Efficacy in Classroom Management, Efficacy in promoting Student Engagement) and Overall Sense of Efficacy. The analysis showed statistically significant differences between age and all factors of TSES; Efficacy in using Instructional Strategies [*F*(2, 255) = 13.20, *p* < 0.001], Efficacy in Classroom Management [*F*(2, 255) = 11.75, *p* < 0.001], Efficacy in promoting Student Engagement [*F*(2, 255) = 7.11, *p* < 0.001], and Overall Sense of Efficacy [*F*(2, 255) = 12.63, *p* < 0.001]. Post-hoc comparisons using the Tukey HSD test were conducted to explore this difference further. For the factor Efficacy in using Instructional Strategies, pairwise comparisons showed that the mean value for the young-adults (*M* = 7.01, *SD* = 0.99) was significantly different from both the mean value of the early-middle-aged adults (*M* = 7.56, *SD* = 0.97) and from the late-middle-aged adults (*M* = 7.71, *SD* = 0.81). There was no statistically significant difference in mean scores between early-middle-aged adults and late-middle-aged adults (*p* = 0.55). For Efficacy in Classroom Management, a post-hoc test showed that the mean value for young-adults (*M* = 6.74, *SD* = 1.26) was significantly different from both the mean value of early-middle-aged adults (*M* = 7.46, *SD* = 1.17) and from late-middle-aged adults (*M* = 7.52, *SD* = 1.08). The mean scores between early-middle-aged adults and late-middle-aged adults showed no statistically significant difference (*p* = 0.94). For the factor Efficacy in promoting Student Engagement, pairwise comparisons showed that the mean value for young-adults (*M* = 6.84, *SD* = 1.08) was significantly different from both the mean value of early-middle-aged adults (*M* = 7.32, *SD* = 1.11) and from late-middle-aged adults (*M* = 7.41, *SD* = 0.92). There was no statistically significant difference in mean scores between early-middle-aged adults and late-middle-aged adults (*p* = 0.83). Lastly, for the factor Overall Sense of Efficacy, post-hoc tests revealed that the mean value for young-adults (*M* = 6.86, *SD* = 1.02) was significantly different from both the mean value of early-middle-aged adults (*M* = 7.45, *SD* = 0.99) and from late-middle-aged adults (*M* = 7.55, *SD* = 0.84). The mean scores between early-middle-aged adults and late-middle-aged adults showed no statistically significant difference (*p* = 0.77). Similar to the results obtained from teachers’ well-being, the results with respect to teachers’ self-efficacy show a general trend where young-adult teachers’ efficacy is different from that of late-middle-aged teachers.

To examine the different predictors of well-being among teachers, a forward stepwise multiple regression analysis was conducted to evaluate how well the variables included in the study predicted teachers’ well-being. The predictor variables were the three levels of TSES (Efficacy in using Instructional Strategies, Efficacy in Classroom Management, Efficacy in promoting Student Engagement) and age, whereas the criterion variable was Overall Well-Being (measured by PERMA).

The stepwise regression equation (Table 3) was statistically significant [*F*(1, 256) = 157.33, *p* < 0.001] for the variance of overall well-being (*R^2^* = 0.38, adjusted *R^2^* = 0.38). Well-being was primarily predicted by efficacy in promoting student engagement.

**Table 3:** Summary of multiple regression for well-being.

Independent predictor variables	*B*	SE	*Β*	*t*	Sig.
Student engagement	0.77	0.06	0.62	12.54	<0.001

## 5. Discussion

The current study aimed to examine teachers’ well-being during a period considered important since it was after the changes that took place due to the COVID-19 pandemic, and the education system was returning to “normality.” Our results show that, at least for the current sample, despite the many changes due to the pandemic, returning to the usual education process did not influence teachers’ well-being. We found that the overall well-being of our sample was moderate to high. This is a significant finding that needs to be further explored. One suggestion is that teachers were able to develop or/and use strategies to overcome the difficulties that arose because of the pandemic and keep their well-being unaffected. That is, even though the pandemic brought many changes in the educational system, with previous research showing that it influences teachers’ well-being (see Kang et al., 2022, for a review), the return to the usual mode of delivery might have enabled teachers to find or utilize strategies to overcome this period and keep their well-being in high levels. Another explanation of this finding lies in the characteristics of the current study’s participants. Recall that almost half of our sample had more than 20 years of experience (46.9%), and almost half had no administrative duties (40.7%). Therefore, it is possible that the moderate to high well-being observed in our current sample is because of these specific characteristics. The current results do not provide clear evidence of which reason is responsible for the observed findings.

The examination of the relationship between teachers’ self-efficacy and well-being showed that all the components of teachers’ self-efficacy (efficacy for using instructional strategies, efficacy in classroom management and efficacy in promoting student engagement) were positively related to all dimensions of the PERMA Profiler (positive emotions, engagement, relationships, meaning, and accomplishment,). This is a significant finding suggesting that teachers’ well-being impacts their teaching practice, their capacity to foster a happy learning environment and their interaction with students and parents. Moreover, teachers’ well-being is influenced by student engagement and a pleasant classroom environment. Our findings are in accordance with the findings of other studies (Arslan, 2018; Kun and Gadanecz, 2019; Turner and Thielking, 2019; Toropova et al., 2020; Skinner et al., 2021; Bartosiewicz et al., 2022; Sun et al., 2022; Han et al., 2023). It appears that a high sense of self-efficacy strengthens teachers’ sense of coping with the difficulties of their work, which might contribute to strengthening their mental health and job satisfaction, which could also strengthen their professional role (Kun and Gadanecz, 2019; Skinner et al., 2021). Furthermore, the current findings suggest that forming a good, positive, supportive, open, and cooperative school climate can improve well-being and teachers’ self-efficacy (Han et al., 2023).

The current findings also suggest that teachers’ well-being is influenced by age. Specifically, we found that young adults (23–39 years old) significantly differ from late-middle-aged adults (52–62 years old) in the two components of the PERMA profiler, meaning and accomplishment. Following PERMA’s explanation of the components, it appears that late middle-aged adults have a clearer purpose and direction in life and a sense of fulfillment and accomplishment than young adults. A possible explanation for this could lie in the promotion process. Primary school teachers, and generally teachers in Greece and Cyprus, throughout their years of employment in schools, reach a point where they seek to be promoted. That is, they can apply to get promoted to principals, vice-principals, educational counselors, and training inspectors. It might be that advancing in their position provides them with a sense of purpose and meaning in life and a sense of accomplishing their personal goals (Seligman, 2011; Symeonidou et al., 2019; Pezirkianidis et al., 2020, 2021). On the other hand, young adults are at the beginning of their teaching career and they might still be trying to get along with their new teacher role; this appears to influence their well-being.

Furthermore, our findings show that teachers’ self-efficacy is also influenced by age. We found that young adults (23–39 years old) showed lower teaching self-efficacy in all three teaching efficacy parameters (instructional strategies, classroom management and student engagement) and overall teaching self-efficacy as compared to early-middle-aged adults (40–51 years old) and late-middle-aged adults (52–62 years old). This finding is supported by previous literature (Billett et al., 2023). The reason behind this finding might lie in young adults teaching experience. As mentioned before, young adults are at the beginning of their careers, and this could influence their sense of self-efficacy. They might still be trying to find effective strategies for providing instructions to students, managing their classroom as well as engaging their students to participate in the lessons. Therefore, supporting the literature, young adults’ ability to organize and deliver their lesson plans, as well as their ability to implement different strategies to handle their classroom, is influenced (Tschannen-Moran et al., 1998).

Importantly, the current study suggests that self-efficacy in promoting student engagement is the most important factor for teachers’ well-being. Even though our current results do not provide a clear indication of how these two are related, the literature that examined remote learning during the period of the COVID-19 pandemic can provide some information regarding their relationship. Specifically, previous research suggests that student engagement was one of the most important variables that have been negatively influenced by remote education (Cardullo et al., 2021). It is not a surprise, therefore, that after returning to the usual mode of education, student engagement changed, which might have resulted in higher self-efficacy in this domain and consequently increased teachers’ well-being. It is imperative for future research to directly examine this relationship.

Notably, this finding also suggests that improving teachers’ self-efficacy in promoting student engagement will positively influence teachers’ well-being. As Fredericks et al. (2004) suggest, student engagement is a multi-dimensional concept that is defined through three main categories: behavioral engagement, emotional engagement, and cognitive engagement. Behavioral engagement includes positive conduct, involvement in learning, participation in school activities, following rules, concentration, attention, persistence, effort, and contribution to class discussions. Emotional engagement includes students’ interest, positive feelings toward the institution and instructors, and feelings of belonging within the institutional environment. Cognitive engagement includes students’ motivational goals and self-regulation learning, metacognition, critical thinking, etc. (Fredericks et al., 2004). There are studies that support that student engagement is correlated with students’ success (Gunuc, 2014; Hao et al., 2018). That is when teachers recognize that they can make their students be actively engaged in the learning process and help their students’ progress in their learning experience, it provides teachers with a sense of fulfillment and satisfaction, which could eventually lead to a boost in their confidence and overall well-being. When teachers feel they are effective in their student’s engagement, they often build strong relationships and emotional connections with them. Feeling connected with their students and knowing that their students could make a difference in their life and succeed could bring a sense of happiness and fulfillment, which in turn, could positively affect teachers’ well-being. Our study suggests the importance of finding and utilizing techniques to improve teachers’ self-efficacy in promoting student engagement, such as communication skills and skills for connecting with their students, among others, that will ultimately improve primary school teachers’ well-being. In this frame, we suggest that teachers attend professional development programs related to such techniques, as they promote the active involvement of students and improve the well-being of teachers.

### 5.1. Limitations

Although this research provides interesting results, it is not without limitations. First, most of the participants were from the Greek population. This limited us from comparing Greek and Cypriot teachers’ well-being. Additionally, the generalizability of the results of the study is also impeded as the education systems in Greece and Cyprus differ as compared to other countries in terms of the qualifications required for teaching in schools, the curriculum, and the structure of the schools. Moreover, we were not able to examine any possible differences between public and private education as most of our participants worked in public schools. Most of our sample had more than 20 years of experience, so we could not study possible differences due to teaching experience. Furthermore, our sample included more females than males. Even though the literature shows that more females than males work in education (Eurostat, 2023), this limited us from making comparisons between the sexes. Teacher reports regarding student engagement, rather than direct observations, and the use of self-report measures are among the limitations identified in the study, which should be considered with caution.

### 5.2. Future research

Based on the limitations of this study, possible suggestions for future research can be made. First, future research would be very interesting to examine the differences between Greek and Cypriot primary school teachers in terms of their self-efficacy and well-being. Furthermore, it will be worth comparing how different environments in public and private schools affect teachers (in their self-efficacy and well-being) and if there are differences in these fields between Greek and Cypriot primary school teachers. Also, it will be interesting to study if teaching experience could affect teachers’ self-efficacy and well-being and if there are differences in these fields between Greek and Cypriot primary school teachers. Lastly, future research should more thoroughly examine the relationship between self-efficacy in promoting student engagement and well-being and strategies that can be used to increase teachers’ self-efficacy in this domain.

## 6. Conclusion

Our results show that self-efficacy has a positive relationship with teachers’ well-being. This is in accordance with the findings of other studies (Arslan, 2018; Toropova et al., 2020; Skinner et al., 2021; Bartosiewicz et al., 2022; Sun et al., 2022; Han et al., 2023). Our results suggest that the most important component of self-efficacy for predicting well-being is efficacy in promoting student engagement. This suggests that developing strategies to promote this domain will positively influence teachers’ well-being. The findings of the current study can be used by educational institutions to create and disseminate professional development courses to increase teachers’ self-efficacy in the domain of student engagement. These courses will aim at strengthening teachers’ self-efficacy in the classroom and equip them with strategies to develop or increase their self-efficacy in student engagement.

## Data availability statement

The raw data supporting the conclusions of this article will be made available by the authors, without undue reservation.

## Ethics statement

This study was approved by the Cyprus National Bioethics Committee (EEBK EΠ 2023.01.11 18.01.2023). The participants provided their informed consent to participate in this study.

## Author contributions

All authors listed have made a substantial, direct, and intellectual contribution to the work and approved it for publication.
